# Supplementary material for: Natural Rubber Composites Using Hydrothermally Carbonized Hardwood Waste Biomass as a Partial Reinforcing Filler—Part II: Mechanical, Thermal and Ageing (Chemical) Properties
Source: Polymers (Basel). 2023 May 21;15(10):2397. doi: 10.3390/polym15102397 (PMC10220732; doi:10.3390/polym15102397)
Supplement: Supplementary file 1 [file polymers-15-02397-s001.zip › polymers-2235390-supplementary.pdf]

SUPPLEMENTARY INFORMATION

Natural Rubber Composites Using Hydrothermally Carbonized Hardwood Waste Biomass as a Partial Reinforcing Filler—Part II: Mechanical, thermal and Ageing Properties

Jelena Lubura [1*], Olga Kočková [2], Beata Strachota [2], Oskar Bera [1], Ewa Pavlova [2], Jelena Pavličević [1*], Bojana Ikonić [1], Predrag Kojić [1] and Adam Strachota [2*]

[1] University of Novi Sad, Faculty of Technology Novi Sad, Bulevar cara Lazara 1, 21000 Novi Sad, Serbia
[2] Institute of Macromolecular Chemistry, Czech Academy of Sciences, Heyrovskeho nam. 2, CZ-162 00 Praha, Czech Republic
* Correspondence: jelenalubura@uns.ac.rs (J.L.); jpavlicevic@uns.ac.rs (J.P.); strachota@imc.cas.cz (A.S.)

1 Thermogravimetric analysis

In Air:

SI-Table S1: TGA in air: Evaluation of residues and steps.

Sample	ZnO (%)	ash (TGA) (%)	ash minus ZnO (%)	CB (%)	HC (%)	ZnO +CB (%)	ash +CB (%)	2nd step (%)	2nd minus ash &CB(* (%)	ZnO +CB +HC (%)	ash +CB +HC (%)	1st+2nd step (%)	1st step (%)	1st+2nd minus [ash &CB &HC](‡ (%)
matrix	3.67	4.75	1.08	0.00	0.00	3.67	4.75	none	n.a.	3.67	4.75	9.10	4.35	4.35
VCB00	2.52	3.53	1.01	0.00	31.45	2.52	3.53	none	n.a.	33.97	34.98	30.50	30.50	-4.48
VCB10	2.52	3.74	1.22	6.29	25.16	8.81	10.03	10.5	0.47	33.97	35.19	30.00	19.50	-5.19
VCB20	2.52	4.25	1.73	12.58	18.87	15.10	16.83	17	0.17	33.97	35.70	34.00	17.00	-1.70
VCB30	2.52	4.26	1.74	18.87	12.58	21.39	23.13	24	0.87	33.97	35.71	35.50	11.50	-0.21
VCB40	2.52	3.65	1.13	25.16	6.29	27.68	28.81	29.5	0.69	33.97	35.10	38.50	9.00	3.40
VCB50	2.52	3.57	1.05	31.45	0.00	33.97	35.02	35.0	-0.02	33.97	35.02	42.00	7.00	6.98

*) physical meaning: additional ash

‡) physical meaning: charred rubber matrix

In Nitrogen:

SI-Table S2: TGA in N2: Evaluation of residues.

Sample	Residue at 900°C (%)	residue other than ZnO(* (%)	CB content (%)	HC content (%)	non-CB & non-ZnO residue (%)	HC carbonized fraction(‡ (% of orig.)
matrix	5.10	2.58	0	0	2.58	n.a.
VCB00	19.30	16.78	0	31.45	16.78	53.4
VCB10	20.30	17.78	6.29	25.16	11.49	45.7
VCB20	24.94	22.42	12.58	18.87	9.84	52.1
VCB30	28.17	25.65	18.87	12.58	6.78	53.9
VCB40	31.61	29.09	25.16	6.29	3.93	62.5
VCB50	34.65	32.13	31.45	0	0.68	n.a.

*) ZnO content: 2.52%

‡) under the simplified assumption, that all the carbonized fraction originates exclusively in HC (not from matrix)

2 Thermo-oxidative aging tests

2.1 Mass losses

SI-Table S3: Weight loss of the natural rubber samples during the thermo-oxidative aging test.

	**Relative mass after specific oxidation time, %**				
	**0 min**	**30 min**	**60 min**	**180 min**	**360 min**
matrix	100	98.95	98.46	97.91	97.55
VCB00	100	97.60	97.21	96.71	96.54
VCB10	100	97.15	98.13	96.60	96.79
VCB20	100	98.11	97.61	96.56	96.92
VCB30	100	97.81	98.26	96.96	98.52
VCB40	100	98.09	98.49	97.67	97.45
VCB50	100	99.60	99.36	98.23	97.25

2.2 DMTA analysis after endured thermo-oxidative aging test time

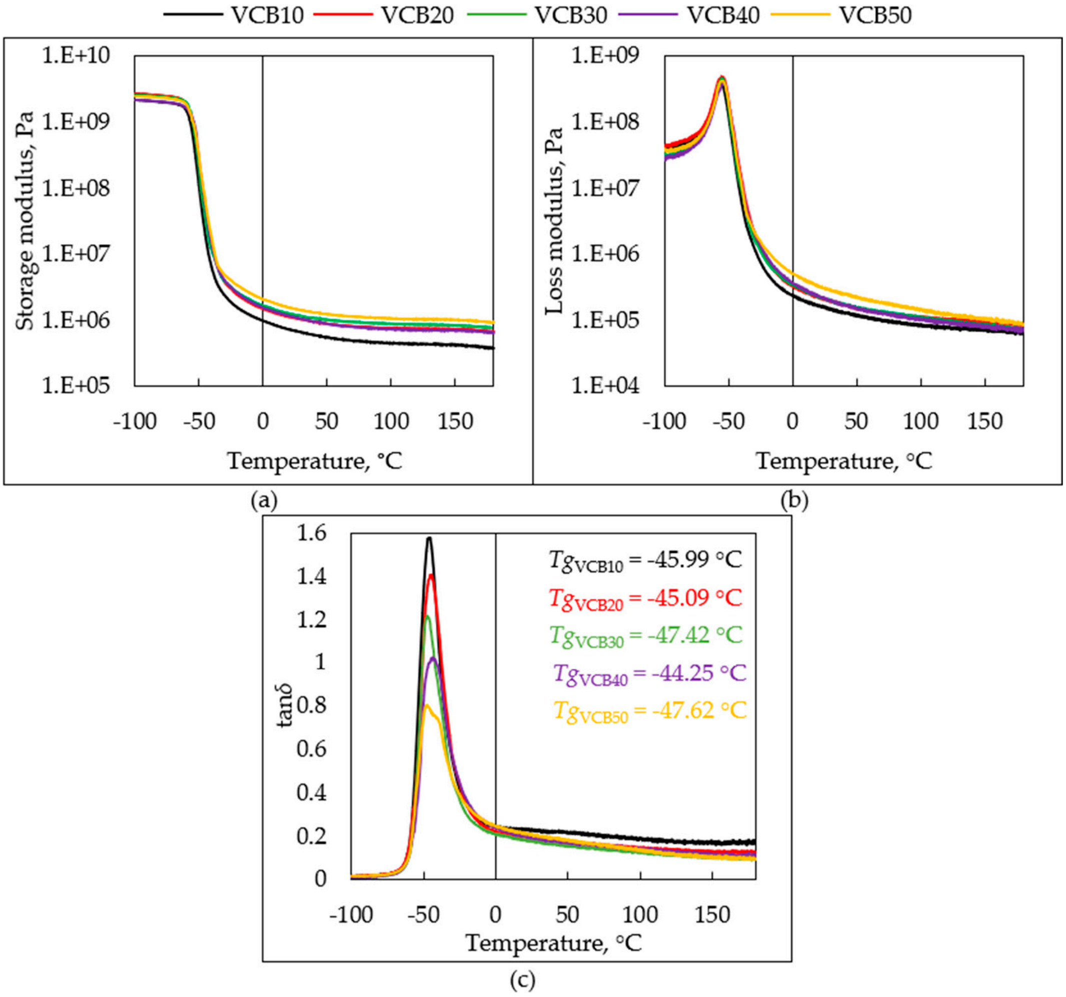

SI-Figure S1. 30 min of oxidation: DMTA analysis: (a) storage modulus $G' = f(T)$; (b) loss modulus $G'' = f(T)$; (c) $\tan\delta$ = f(T).

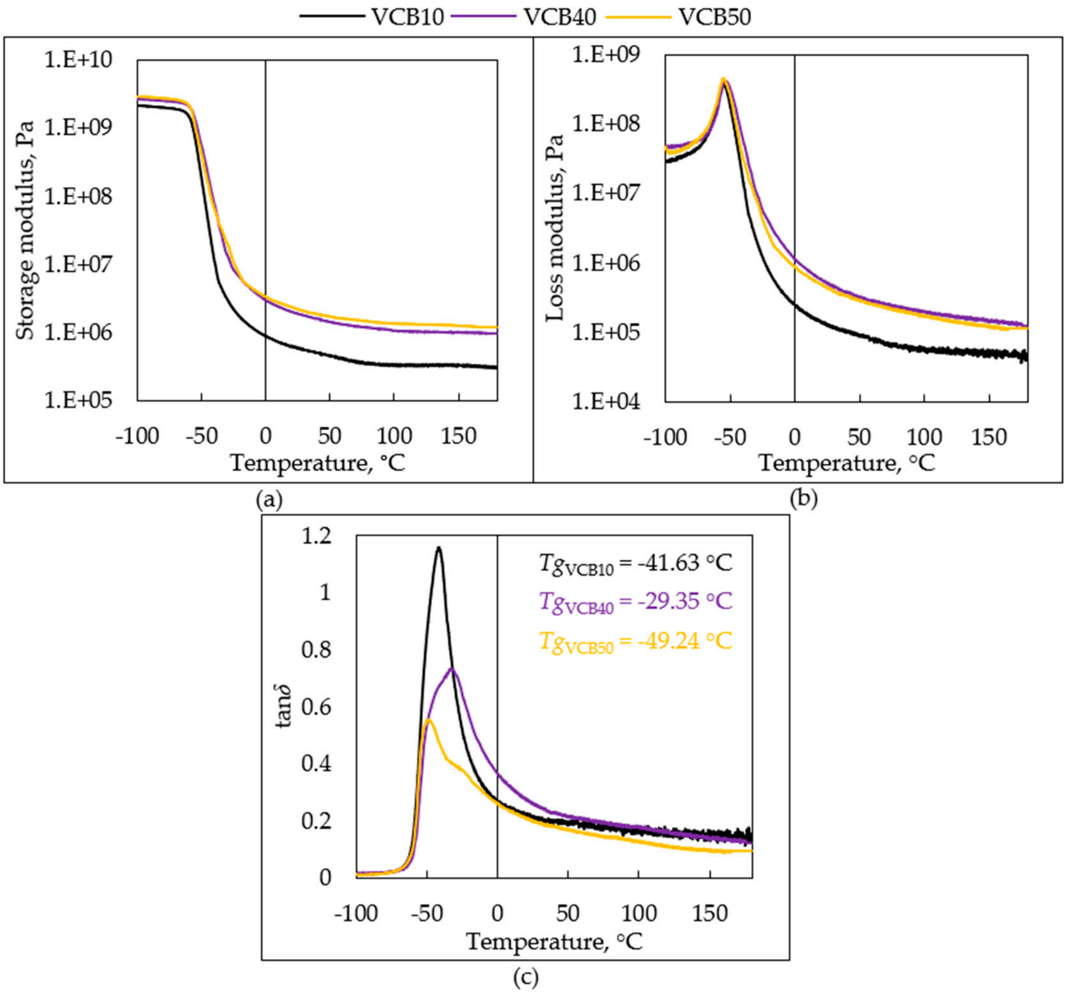

SI-Figure S2. 60 min of oxidation: DMTA analysis: (a) storage modulus $G' = f(T)$; (b) loss modulus $G'' = f(T)$; (c) $\tan\delta = f(T)$.

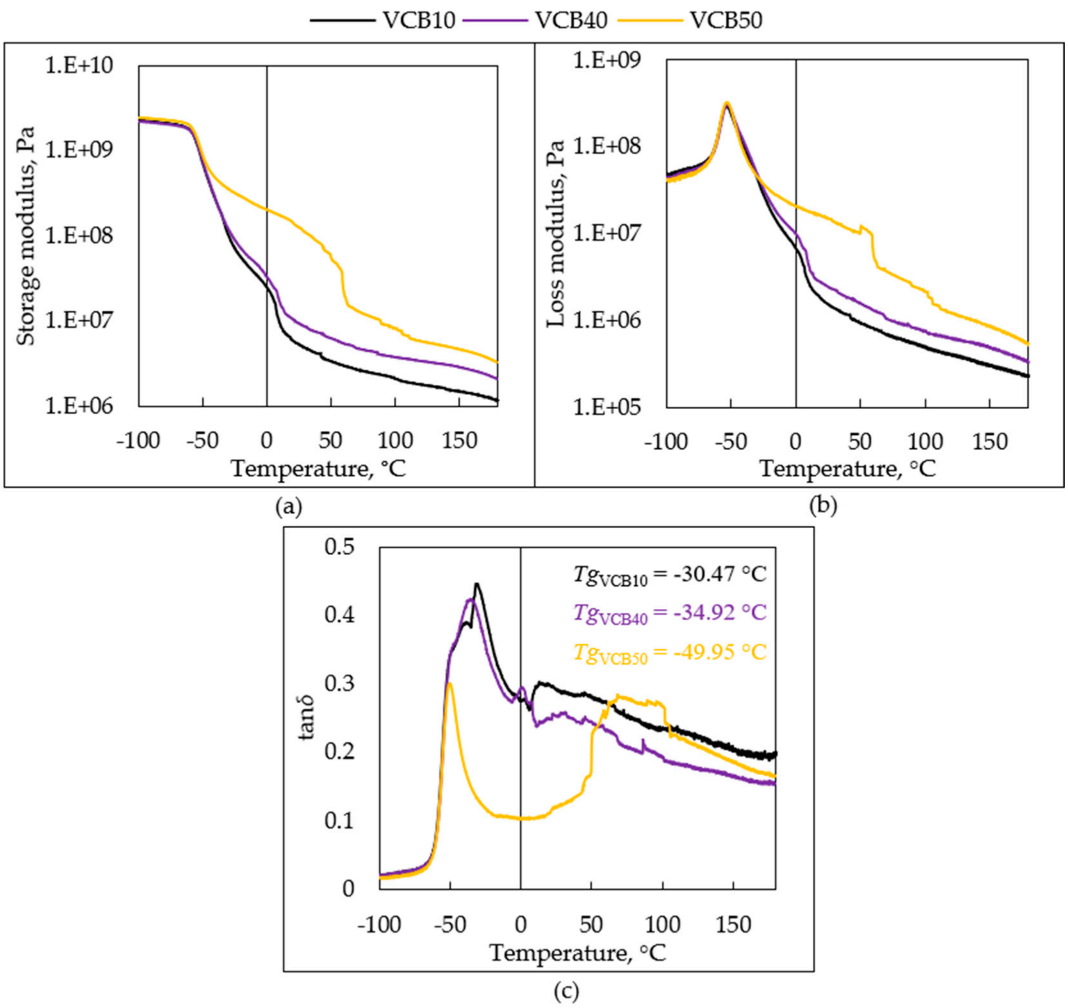

SI-Figure S3. 360 min of oxidation: DMTA analysis: (a) storage modulus $G' = f(T)$; (b) loss modulus $G'' = f(T)$; (c) $\tan\delta = f(T)$.

3 Florida tests

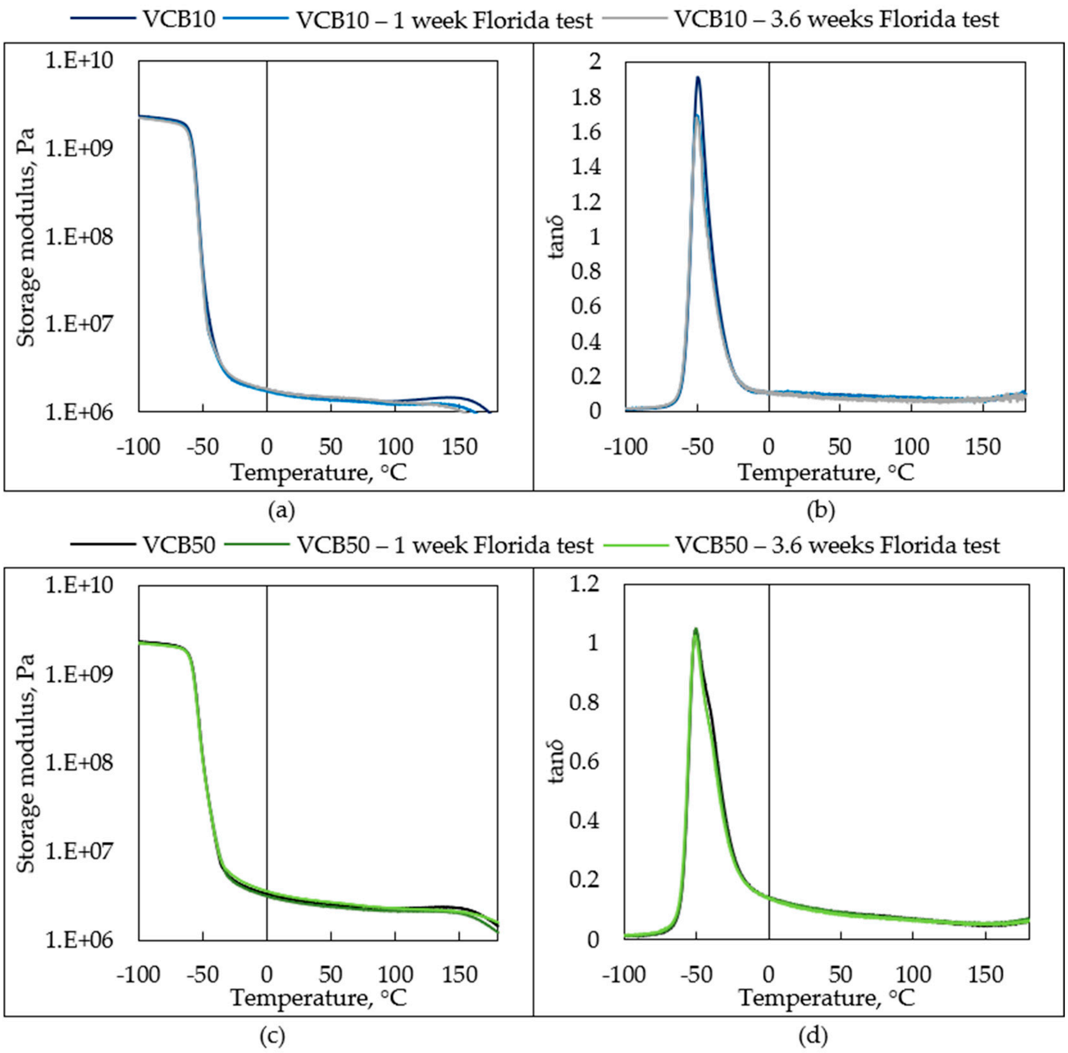

SI-Figure S4. DMTA results for the samples (a, b) VCB10 and (c, d) VCB50, in the intact state, after 7 days, and after 25 days of aging in the Florida test (different line colors); the duration of the Florida tests simulates 2¾ and 10 months of aging in hot and humid climate, respectively; (a, c) $G' = f(T)$; (b, d) $\tan\delta = f(T)$.

<u>**Trends in tan*δ***</u>

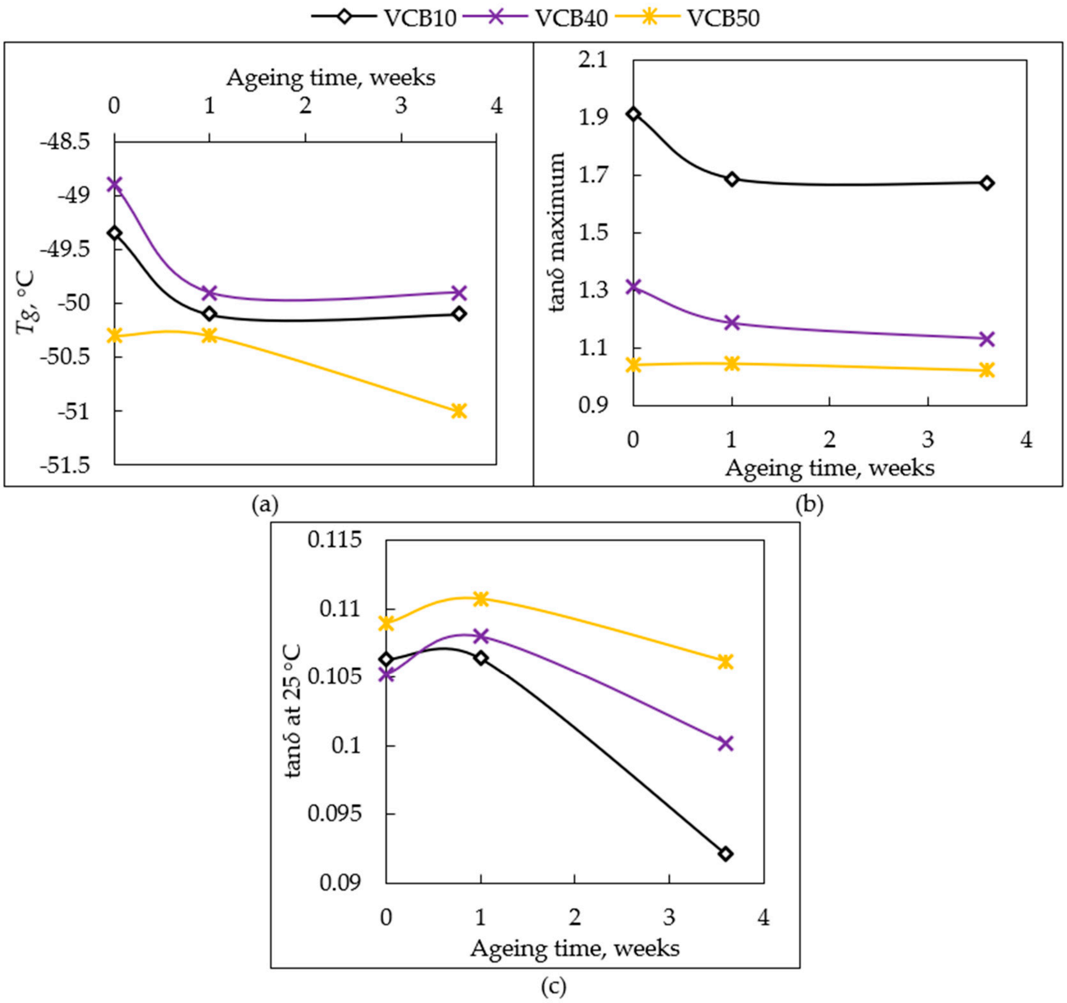

SI-Figure S5. Trends in simulated aging (Florida tests) for the samples VCB50 (no hydrochar), VCB40, and VCB10: (a) slight shifts of the glass transition temperature T_g in dependence on the aging time; (b) modest changes in the height of tan*δ* peak at T_g, in dependence on the aging time; (c) slight changes in the tan*δ* value at 25°C, in dependence on the aging time; note: the aging times of 1 and 3.6 weeks simulate 2¾ and 10 months of aging in hot and humid climate.
